# Insights into knee post-traumatic osteoarthritis pathophysiology from the relationship of serum biomarkers to radiographic features in the ADVANCE cohort

**DOI:** 10.1186/s13075-025-03648-y

**Published:** 2025-11-05

**Authors:** Oliver O’Sullivan, Ana M. Valdes, Fraje Watson, Stefan Kluzek, Anthony M. J. Bull, Alexander N. Bennett

**Affiliations:** 1 Academic Department of Military Rehabilitation (ADMR), Defence Medical Rehabilitation Centre (DMRC), Stanford Hall, Loughborough, UK; 2 Academic Unit of Injury, Recovery and Inflammation Sciences, Faculty of Medicine and Health Sciences, University of Nottingham, Nottingham, UK; 3 Nottingham NIHR Biomedical Research Centre, Faculty of Medicine and Health Sciences, University of Nottingham, Nottingham, UK; 4 Department of Twin Research & Genetic Epidemiology, King’s College London, London, UK; 5 Department of Bioengineering, Imperial College London, London, UK; 6 Centre for Sport, Exercise and Osteoarthritis Research Versus Arthritis, University of Nottingham, Nottingham, UK; 7 Centre for Blast Injury Studies, Department of Bioengineering, Imperial College London, London, UK; 8 National Heart and Lung Institute, Imperial College London, London, UK

**Keywords:** Pathophysiology, Serum biomarkers, Anabolism, Catabolism, Inflammation, Post-traumatic osteoarthritis

## Abstract

**Introduction:**

Post-traumatic osteoarthritis (PTOA) is a complex condition with multiple pathological processes at play. Molecular biomarkers can enable a better understanding of these processes, thus enhancing case endotyping, phenotyping, personalised care and drug discovery. The longitudinal ArmeD SerVices TrAuma and RehabilitatioN OutComE (ADVANCE) study offers the opportunity to develop insights into PTOA pathophysiology using a panel of extra-cellular matrix turnover, inflammatory and metabolic biomarkers and their cross-sectional (associative) and longitudinal (predictive) relationship to features of radiographic PTOA in a cohort of young, male, severely injured servicepersonnel.

**Methods:**

Using serum and radiographic data gathered in the baseline (8-years) and first follow-up visit (11-years) post-injury of ADVANCE (*n* = 1145), two analyses were undertaken. Firstly, cross-sectional univariate analysis between serum COMP, CTX-II, PIIANP, IL-1b, IL-17a, TNF-a, leptin and adiponectin and radiographic features (joint space narrowing (JSN), osteophytes and sclerosis), followed by the longitudinal prediction of new or progression of these three radiographic features using LASSO to select predictors. The area under a ROC curve (AUROC) was computed.

**Results:**

Complete radiographic and serum case data in *n* = 872 male British servicemen, aged 34.5 (5.5) at baseline and 38.3 (5.4) at follow-up were analysed. Those with JSN had significantly higher concentrations of leptin (FDR-corrected q-value, q = 0.04). COMP had an AUROC of 0.604 (0.543,0.664) for new cases of JSN, COMP, IL-1β and leptin had an AUROC 0.586 (0.524,0.646) for new osteophytes, and TNF-α, IL-1β and adiponectin had an AUROC 0.590 (0.520,0.659) for new sclerosis.

**Conclusion:**

This large, unique study suggests different pathological processes underpinning each radiographic feature of PTOA, including predominant unbalanced ECM-catabolism and inflammation contributing to JSN, ECM-catabolism and increased inflammation contributing to osteophyte development and an inflammation-predominant process contributing to subchondral sclerosis.

**Supplementary Information:**

The online version contains supplementary material available at 10.1186/s13075-025-03648-y.

## Introduction

Osteoarthritis is a complex, multifaceted disease influenced by modifiable and non-modifiable risk factors, resulting in a clinical continuum from a prodromal asymptomatic condition to a highly disabling painful clinical syndrome [1]. It has been estimated that 13% of knee OA can be attributed to previous trauma [2], with certain injuries, such as anterior cruciate ligament (ACL) rupture, meniscal injury, or intra-articular fracture, carrying a significant risk of subsequent post-traumatic OA (PTOA), presenting within a few years after injury, with a period of intermittent symptomatic recovery prior to symptomatic and functional decline [3–5].

The initial traumatic episode can result in localised disruption of articular cartilage, cartilage fissures, and chondrocyte death, accompanied by a post-traumatic inflammatory response and synovitis, as well as concurrent damage affecting the biomechanical function of the joint, such as fractures [6]. Immediately after this, multiple signalling pathways activate, resulting in cartilage matrix degradation and synovial inflammation, leading to repair, remodelling, and adaptation. However, aberrant mechanistic pathways can contribute to a lack of repair and remodelling, inadequate adaptation, and the subsequent development of PTOA, with altered biomechanics, metabolism, and low-grade inflammation resulting in disturbed joint homeostasis. Unlike idiopathic OA, where pathological articular cartilage changes trigger negative surrounding structural changes, it is possible that in early PTOA, structural changes influence local bone and cartilage compositional changes [3, 6–8]. These changes involve cartilage matrix macromolecule synthesis or subchondral bone resorption, mediated by cytokines, and leave signals which can be detected at a molecular level [9].

Certain molecular biomarkers relate to future pain or disease progression, with academic-regulatory-pharmacological cross-sector coalescence around the most promising biomarkers to improve their validation and qualification spearheaded by the National Institute of Health [10, 11]. Phase 2 of this work, the OA PROGRESS study, is nearing completion, with the ambition to enable the validation of biochemical (and imaging) measures for prognosis and prediction of disease progression over 48 months using thousands of pooled participant data from existing trial data [12]. However, the vast majority of those included in these pooled data are aged ≥ 60 years old [12], and whilst OA does become more common with age, it can commence and present during any stage of adult life, especially in those with a traumatic cause. In particular, in nearly 40% of those with OA, symptoms commenced before 45-years-old, with those experiencing symptoms prior to 35 years old potentially waiting a decade before receiving a diagnosis [13].

Certain occupational groups, such as physical jobs, sportspeople and emergency services, are associated with an increased risk of PTOA [14–16]. Additionally, after exposure to major trauma, PTOA can present far sooner, in less than two years [3]. The Armed Services Trauma Rehabilitation Outcome (ADVANCE) study is a prospective, longitudinal cohort study which aims to understand the long-term physical and psychosocial effects of combat injury in 1145 British servicemen, half of whom were seriously injured during the Afghanistan war. The ADVANCE study offers a young, heterogenous and high-risk cohort to understand the relationship between molecular biomarkers and OA pathophysiology [17]. Earlier work demonstrated increased rates of knee OA across the cohort, two-fold in those with any injury increasing to 4x in those with combat injury or lower-limb loss [18], but also nearly 20% of the non-injured comparison British servicemen [19]. Although serum biomarker analysis were not able to differentiate those with and without OA, it did identified that there was no molecular difference between those with OA in the exposed group (PTOA) and in the unexposed group (early-onset idiopathic OA) [19].

Other cohorts have demonstrated an increased post-injury risk, plateauing as time from injury increases [16, 20], a hypothesis supported in the ADVANCE cohort by no increased rates of radiographic OA between exposed and unexposed groups between the Baseline visit (8 years from injury) and the first Follow-up visit three years later (11 years from injury) [21]. Therefore, the ADVANCE study offers an exciting opportunity to further explore the pathophysiological processes of OA present in a younger cohort, using the individual components of the Osteoarthritis Research Society International (OARSI) radiological atlas (joint space narrowing (JSN), osteophytes, sclerosis) [22] and panel of extracellular matrix turnover, inflammatory and metabolic serum biomarkers. This unique cohort will enable the opportunity to elucidate the natural course of the disease, and add to the evidence base for the further quantification of established serum biomarkers.

The hypothesis of this study is that serum biomarkers measuring different pathophysiological processes will be associated and predictive of different radiological features of PTOA. The aim is two-fold; firstly, to undertake a cross-sectional analysis of the panel of candidate serum biomarkers to OARSI radiographic atlas features; and secondly, to undertake a longitudinal analysis to ascertain the ability of these biomarkers to predict individual radiological feature development or progression over three years.

## Methods

### Ethics

Favourable opinion for ADVANCE was granted by MoD Research Ethics Committee (MODREC:357PPE12) with subsequent approval from the University of Nottingham Faculty of Medicine and Health Sciences REC (UoN FMHS 170–1122). Study participation is voluntary, with written informed consent from each participant at each study visit, with the study performed in accordance with the Declaration of Helsinki.

### PPI

Public and Patient Involvement is undertaken using a variety of methods, including face-to-face and remote participant panels, feedback questionnaires for each study visit, newsletters, participant-focussed impact reports, and the study website (www.advancestudydmrc.org.uk). These have helped refine study design, logistics and tailor further research questions relevant to the interests of the participants.

### Participants

ADVANCE is a longitudinal cohort study monitoring the long-term physical and psychosocial outcomes following combat injury in UK military personnel who served in Afghanistan, involving 579 participants who sustained combat injuries requiring aeromedical evacuation to the UK, and 566 participants not exposed to combat injury who were frequency-matched for age, rank, role, service, and deployment [17]. Further details on injuries can be found here [19]. Female military personnel were excluded due to the number sustaining combat injuries being too low to generate sufficient statistical power. Additional information on recruitment and inclusion/exclusion criteria (including acute active infection) can be found in the protocol paper and previously published papers [17–19]. Participants were identified using military health records, with frequency matched comparison participants identified by the Defence Statistics (Health) team.

### Data collection

Study visits occurred at the Defence Medical Rehabilitation Centre Headley Court (2015–2018) or Stanford Hall (2018–2024) over the period of one day, with participants fasted and absent from caffeine and alcohol for at least 8 h before the visit. Trained research nurses collect a range of assessments, including sociodemographic, anthropometric, medical history (including existing medical problems, combat trauma, and significant family history), serum sampling, and radiographs of their knees. Study data were collected and managed using Research Electronic Data Capture (REDCap), a secure, web-based software platform [23]. This study reports data collected at the Baseline (8-years post-injury/deployment) and first Follow-up (11-years post-injury/deployment visit of the ADVANCE study.

### Radiography

Semi-flexed (7–10°) posterior-anterior views of all possible participant knees were taken using a Synaflexer X-ray positioning frame (Synarc Inc, San Francisco, California). The tibiofemoral joint was scored using the OARSI atlas [22], using KOALA with manual checking, which grades JSN, sclerosis and osteophytes using a 4-point scale (0 – none, 1 – mild, 2 – moderate, 3 – severe). No member of the radiographic reporting team was aware of the participant’s clinical status. The FDA-approved Knee OA Labelling Assistant (KOALA, Image Biopsy Lab, Vienna, Austria) with manual checking scored the x-ray, offering an accuracy of 76%, 84%, 60%, sensitivity of 97%, 88%, 97% and specificity of 41%, 73% and 24% for all grades of JSN, osteophyte and sclerosis (≥ 1) [24]. The aided AI KOALA tool improves reader agreement rates by 1.37, 1.59, and 1.42-fold when assessing JSN, osteophyte or sclerosis grade [25].

Analyses were performed using the presence of any individual measures of the OARSI atlas (JSN, osteophytes, sclerosis) to ascertain if any specific biomarkers offered improved identification of different pathological mechanisms in the cartilage, surface or subchondral bone matrix. When participants had two variables (from both knees), the index knee score signifying more advanced rOA was selected (higher JSN/osteophyte/sclerosis grade). Progression was defined as an increase in individual radiographic feature (JSN/osteophyte/sclerosis) by ≥ 1 at Follow-up in a knee with at least JSN/osteophyte/sclerosis ≥ 1 at Baseline. Incidence was defined as the presence of a radiographic feature of knee OA (JSN/osteophyte/sclerosis ≥ 1) at Follow-up in a knee that was JSN/osteophyte/sclerosis 0 at Baseline. Each feature was taken in turn and analysed separately to understand if there was a relationship between the serum biomarker and the possible pathophysiological change reflected by the individual radiographic feature.

### Biomarker analysis

Participants underwent serum sampling with fasted blood taken from the antecubital fossa using the Vacutainer system. It was centrifuged at 3500 rpm for 10 min, and then aliquoted to be stored into cryovials and stored in monitored freezers at -80°. Frozen baseline samples were analysed by Affinity Biomarker Laboratory (ABL), London, for cartilage oligomeric matrix protein (COMP), C-terminal cross-linked telopeptide of type II collagen, (CTX-II), N-propeptide of collagen IIA (PIIANP), interleukin (IL)-1b, IL-17a, tumour necrosis factor (TNF)-a, leptin and adiponectin via meso scale discovery (MSD) or enzyme-linked immunosorbent assay (ELISA). This panel of serum candidate biomarkers, only sampled at baseline, were chosen to examine different pathological mechanisms, including aberrant tissue turnover, inflammatory dysfunction and metabolic dysregulation.

Results underwent quality assurance and control by ABL, who were also not aware of the participants clinical status, using three internally identified quality control samples and two kit controls. The worst reported intra- or inter-variability coefficient of variation for each biomarker were; MSD: IL-17a < 9.5%, IL-1β < 7%, TNF-a < 15%; ELISA: COMP < 12%, leptin < 7%, adiponectin < 8%, CTX-II < 11%, PIIANP < 6%. Any biomarker concentration level below the lower limit of quantification (LLOQ) was given a value halfway between zero and the LLOQ threshold, with those above the upper LOQ (ULOQ) given ULOQ threshold + 1, required for IL-17a (< 0.54 = 0.27, *n* = 24), PIIANP (< 5.9 = 2.95 *n* = 23, > 1000 = 1001, *n* = 9), CTX-II (< 0.1 = 0.05, *n* = 421), and IL-1β (< 0.043 = 0.0215, *n* = 713).

### Statistical analysis

All data were screened for normality visually using histograms, with parametric and non-parametric testing used accordingly and presented as mean (standard deviation, SD) and median (interquartile range, IQR), respectively. As a result of earlier work, demonstrating no molecular differences between those with OA [19], participants are dichotomised due to presence of individual radiographic OA feature. The analysis was performed in two parts, to address each aim in turns.

First, an initial descriptive analysis was performed to identify presence and amount of individual OA features, with correlation analysis performed with biomarkers standardised to a mean = 0 and SD = 1 to visualise patterns in the data (Spearman’s or Pearson’s, full results in Supplementary File 1). Univariate analysis was subsequently performed, depending on normality and the presence or absence of each individual radiographic feature (Wilcoxon rank sum and Student’s t test). Unadjusted analyses were initially performed using the baseline data, followed by adjusted, with the confounders age, body mass, time from injury/deployment, exposure to trauma, military rank (as a proxy for socio-economic status, SES [26, 27]) and ethnicity adjusted for. This was performed by transforming the biomarkers using their natural logarithm and adjusted for the confounders using a regression model, with studentised residuals created and taken forward for analysis. Within an athletic population, the body mass index (BMI) can ‘overscore’ individuals with a high muscle mass; therefore, a body shape index (ABSI) [28], calculated with BMI and waist circumference, was utilised in this analysis rather than BMI, which gives a more balanced reflection of body weight and central adiposity. Time from injury/deployment was measured from the participant’s index deployment.

Next, using longitudinal data from the ADVANCE 11-year follow up visit, an analysis of the predictive value of the serum biomarkers was performed. Participant x-rays were assessed and dichotomised for either the incidence or progression of each individual radiographic feature as described above, with correlation analysis again used with standardised biomarkers to visualise patterns (full results, Supplementary File 1). Due to the numbers of participants within each of these groups, a least absolute shrinkage and selection operator (LASSO) variable selection model was performed to identify significant relationships between serum biomarkers and radiographic features. When significant predictors were identified, multivariate logistic regression was performed, with Nagelkerke’s R2 and area under the receiver operator curve (AUROC) reported (further regression results in Supplementary File 2). Due to the small numbers in groups, full adjustment was not possible, with only unadjusted results reported.

The ADVANCE study recruited *n* = 1145 male British servicemen (trauma-injured *n* = 579, comparison *n* = 566) at baseline, with *n* = 1052 attending the first follow-up visit (92%). A whole case approach was adopted for this analysis, therefore, the *n* = 190 participants who did not have x-ray at both time points or biomarker data available were excluded, inclusive of those who did not attend follow-up (*n* = 93). In addition, to prevent confounding due to the likely different pathological processes at play for those sustaining lower-limb loss and subsequent OA [29], the *n* = 161 individuals sustaining amputations were also excluded, leaving 872 participants.

Given the large number of comparisons performed in this exploratory study, results have been additionally corrected using the Benjamini and Hockberg method, with results corrected using the false discovery rate (q-value) [30], with the significance rate set at 0.1. Data is presented with the unadjusted, uncorrected *p*-value and the fully adjusted, corrected q-value. Presenting data both in its unadjusted and adjusted forms allows other studies to compare results and enables the effect of OA to be more accurately partitioned. Analyses were performed in Stata 18.5 (StataCorp LLC, Texas) and GraphPad Prism 10 (Dotmatics, Boston).

## Results

Eight-hundred and seventy-two male participants, aged 34.5 (5.5) at the Baseline and 38.3 (5.4) at the Follow-up visit are included, at a mean 9.0 (2.2) years from injury (for the 42% of injured participants) or index deployment at Baseline and 3.3 years (0.6) between Baseline and Follow-up (Table 1).

**Table 1:** Participant demographics

	Total (*n* = 872)
Age, yrs (BL)	34.5 (5.5)
Age, yrs (FU)	38.3 (5.4)
BMI, kg/m^2^ (BL)	27.6 (3.5)
BMI, kg/m^2^ (FU)	28.2 (3.7)
Abdo. circum., cm (BL)	93.0 (87.5-100.5)
Abdo. circum., cm (FU)	96.0 (90.0-103.0)
Exposed, n= (%)	364 (41.7%)
Military Rank, n= (%)	
Junior NCO	542 (62%)
Senior NCO	211 (24%)
Officer	119 (14%)
Time from injury, yrs	9.0 (2.2)
Time to follow-up, yrs	3.3 (0.6)
White ethnicity, n= (%)	771 (88.5%)

BL: Baseline, FU: Follow up, yrs: years, BMI: body mass index, kg: kilograms, M: metres, abdo circum: abdominal circumference, cm – centimetres, NCO – non-commissioned officer. Data presented as mean ± standard deviation, median (interquartile range) or *n*= (%)

At Baseline, *n* = 331 (38%) participants had JSN, *n* = 165 (19%) had osteophytes and *n* = 74 (9%) sclerosis evident on their knee radiographs, rising to *n* = 370 (42%), *n* = 233 (27%) and *n* = 117 (13%) by Follow-up, respectively (Table 2). Across all categories, the vast majority were Grade 1 (Table 2), with a mild increase in severity between study visits, with 3% of JSN, 10% of osteophyte and 16% of sclerosis cases increasing by ≥ 1 grade (Table 3). In individuals without specific radiological features at Baseline, 19% developed new JSN, 15% developed new osteophytes and 9% developed new sclerosis by Follow-up (Table 3).

**Table 2:** Rates of joint space narrowing (JSN), osteophytes and sclerosis in the ADVANCE cohort at baseline and follow-up

	Baseline	Follow-up
JSN, *n*= (%)	331 (38.0%)	370 (42.4%)
JSN grade, *n*= (%)		
0	541 (62%)	502 (58%)
1	307 (35%)	343 (39%)
2	21 (2%)	20 (2%)
3	3 (0%)	7 (1%)
Osteophytes, *n*= (%)	165 (18.9%)	233 (26.7%)
OP grade, *n*= (%)		
0	707 (81%)	639 (73%)
1	132 (15%)	191 (22%)
2	16 (2%)	19 (2%)
3	17 (2%)	23 (3%)
Sclerosis, *n*= (%)	74 (8.5%)	117 (13.4%)
Scl grade, *n*= (%)		
0	798 (92%)	755 (87%)
1	64 (7%)	98 (11%)
2	10 (1%)	18 (2%)
3	-	1 (0%)

JSN: Joint space narrowing, Scl: Sclerosis, OP: Osteophytes. Data presented as *n*= (%)

**Table 3:** Rates of new or progressive joint space narrowing (JSN), osteophytes and sclerosis between baseline and follow-up

JSN		Osteophytes		Sclerosis	
New	Progress	New	Progress	New	Progress
105/541 (19%)	10/331 (3%)	104/707 (15%)	17/165 (10%)	69/798 (9%)	12/74 (16%)

**Fig. 1:**
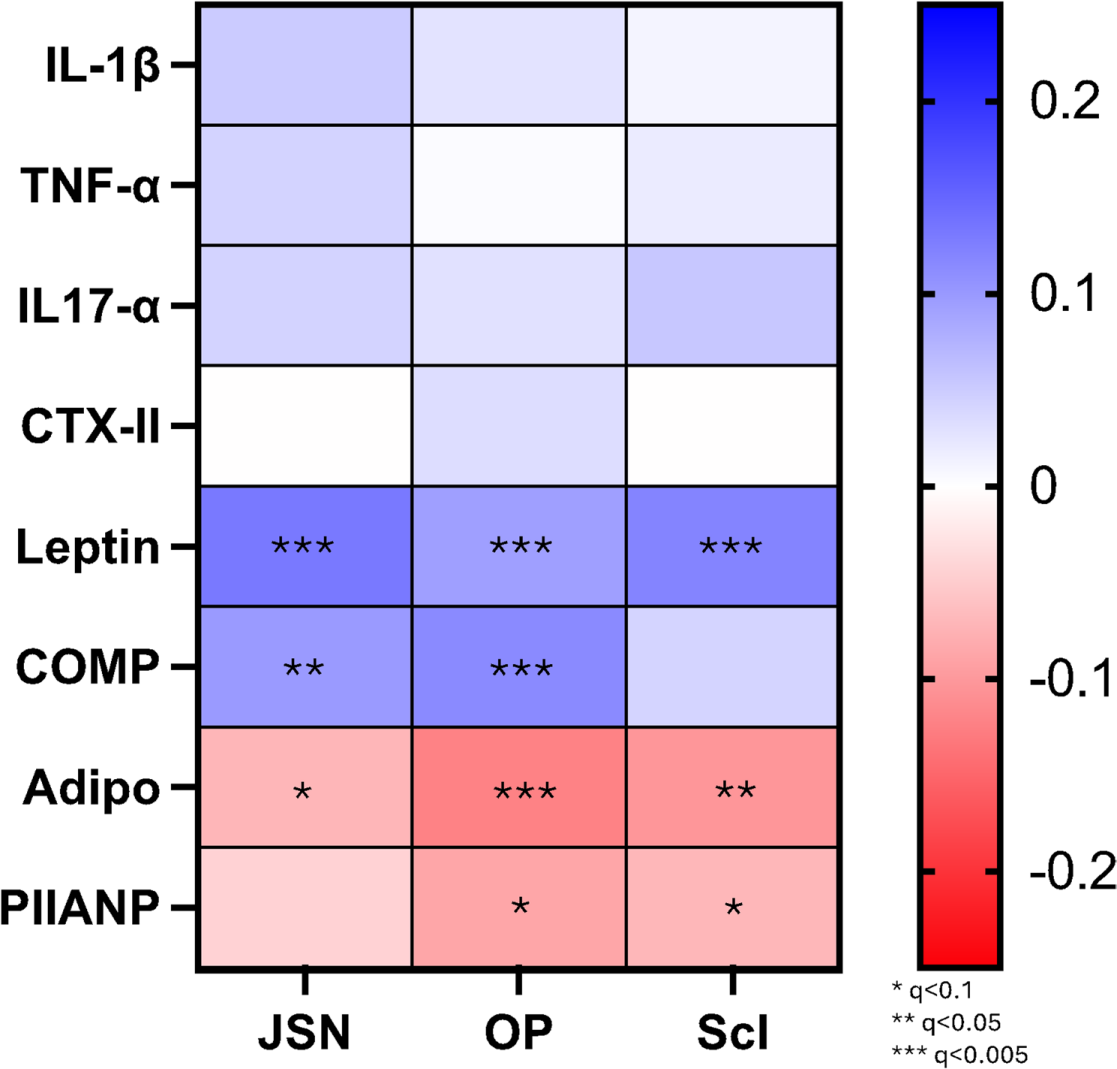
Cross-sectional correlations between the panel of serum biomarkers and individual radiographic features (joint space narrowing, osteophyte, sclerosis). *Correlations are corrected for using the false discovery rate (q-value). All investigations at baseline (8-year) visit*. *IL: Interleukin*,* TNF: Tumour necrosis factor*,* CTX-II: C-terminal cross-linked telopeptide of type II collagen*,* COMP: cartilage oligomeric matrix protein*,* PIIANP: N-propeptide of collagen IIA*,* Adipo: Adiponectin. JSN: Joint space narrowing*,* OP: osteophytes*,* Scl: Sclerosis*

Figure 1 shows the cross-sectional correlation between the panel of serum biomarkers and individual radiographic features performed at Baseline (full results, Supplementary File 1). Those with JSN features at Baseline had significantly higher levels of COMP (*p* = 0.002) and leptin (*p* < 0.001) (Table 4). After adjustment and correction, the difference in leptin remained significant (q = 0.04). COMP (*p* < 0.001) and leptin (*p* = 0.006) were significantly higher, and PIIANP significantly lower (*p* = 0.009) between those with and without osteophytes at Baseline; however, after adjustment and correction, no differences remained significant (Table 4). In those with sclerosis at Baseline, leptin was higher (*p* < 0.001), with PIIANP (*p* = 0.036) and adiponectin (*p* = 0.015) both significantly lower than those without (Table 4). After adjustment and correction, neither remained significant.

**Fig. 2:**
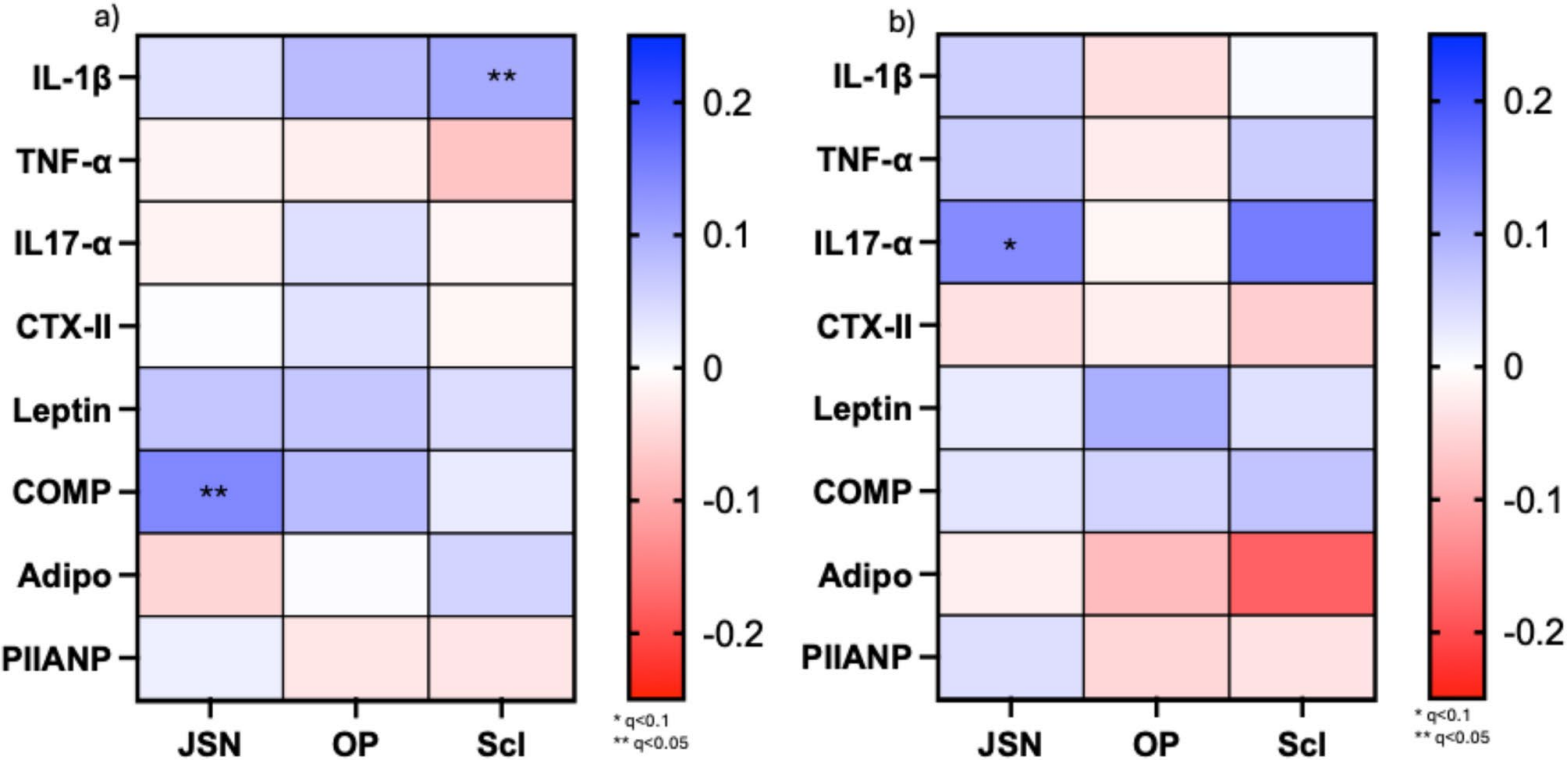
Longitudinal correlations between the panel of serum biomarkers and new (**a**) or progression of (**b**) radiographic features (joint space narrowing, osteophyte, sclerosis). *Correlations are corrected for using a false discovery rate (q-value). Serum biomarkers from 8-year visit*,* radiographs from 11-year visit. IL: Interleukin*,* TNF: Tumour necrosis factor*,* CTX-II: C-terminal cross-linked telopeptide of type II collagen*,* COMP: cartilage oligomeric matrix protein*,* PIIANP: N-propeptide of collagen IIA*,* Adipo: Adiponectin. JSN: Joint space narrowing*,* OP: osteophytes*,* Scl: Sclerosis*

Figure 2 shows the longitudinal correlation between the panel of serum biomarkers and the new or progressive cases of the individual radiographic features (full results, Supplementary File 1). LASSO selected COMP as a predictor for new cases of JSN, with an area under the receiver curve (AUROC) of 0.604 (95% confidence interval, CI, 0.543,0.664) and R2 0.018 (Supplementary File 2). COMP, IL-1β and leptin were selected as predictors of new osteophyte cases, with an AUROC = 0.586 (95% CI 0.525,0.646), R2 0.018 (Supplementary File 2). TNF-α, IL-1β and adiponectin were selected as predictors for new cases of sclerosis, AUROC = 0.590 (95% CI 0.520,0.659), R2 0.022 (Supplementary File 2). No biomarkers were selected by LASSO for the prediction of JSN, osteophyte or sclerosis progression.

**Table 4:** Serum biomarker concentrations in those with and without joint space narrowing, osteophytes and sclerosis within the ADVANCE cohort

	Total	No JSN	JSN	*p*-value *q*-value	No OP	OP	*p*-value *q*-value	No Scl	Scl	*p*-value *q*-value
	*N* = 872	*N* = 541	*N* = 331		*N* = 707	*N* = 165		*N* = 798	*N* = 74	
IL-1β	0.02 (0.02–0.06)	0.02 (0.02–0.06)	0.02 (0.02–0.06)	0.713~ 0.811~	0.02 (0.02–0.06)	0.02 (0.02–0.05)	0.340~ 0.402~	0.02 (0.02–0.06)	0.02 (0.02–0.05)	0.490~ 0.678~
TNF-α	1.94 (0.61)	1.92 (0.60)	1.97 (0.62)	0.280# 0.527#	1.95 (0.64)	1.92 (0.46)	0.640# 0.959#	1.94 (0.62)	1.94 (0.46)	0.980# 0.825#
IL17-α	1.27 (0.97–1.79)	1.26 (0.96–1.71)	1.31 (0.97–1.87)	0.210~ 0.512#	1.27 (0.96–1.78)	1.30 (1.00-1.86)	0.400~ 0.690#	1.27 (0.96–1.78)	1.38 (1.09–1.86)	0.106~ 0.794#
CTX-II	0.21 (0.05–0.64)	0.20 (0.05–0.65)	0.21 (0.05–0.64)	0.700~ 0.512~	0.21 (0.05–0.64)	0.20 (0.05–0.63)	0.450~ 0.690~	0.20 (0.05–0.63)	0.32 (0.05–0.72)	0.390~ 0.828~
Leptin	5.59 (2.99–9.04)	5.08 (2.52–8.44)	6.40 (3.59–9.67)	**< 0.001~** **0.04~**	5.36 (2.88–8.85)	6.26 (4.00-10.12)	**0.006~** 0.402~	5.44 (2.88–8.86)	7.02 (4.31–11.52)	**< 0.001~** 0.108~
COMP	273.90 (87.73)	266.77 (85.28)	285.55 (90.51)	**0.002#** 0.512#	268.77 (85.66)	295.87 (93.19)	**< 0.001#** 0.402#	273.02 (88.00)	283.40 (84.66)	0.330# 0.825#
Adipo	6.43 (4.60)	6.54 (4.44)	6.27 (4.84)	0.400# 0.512#	6.56 (4.54)	5.90 (4.79)	0.098# 0.402#	6.55 (4.73)	5.19 (2.39)	**0.015#** 0.108#
PIIANP	110.50 (74.30-163.95)	114.20 (74.90-167.10)	103.90 (73.70-159.40)	0.190~ 0.977#	113.30 (78.40-167.30)	97.20 (65.20-151.90)	**0.009~** 0.402#	111.75 (75.30-164.90)	91.60 (64.80-153.70)	**0.036~** 0.678#

IL: Interleukin, TNF: Tumour necrosis factor, CTX-II: C-terminal cross-linked telopeptide of type II collagen, COMP: cartilage oligomeric protein, PIIANP: N-propeptide of collagen IIA, Adipo: Adiponectin. JSN: Joint space narrowing, OP: osteophytes, Scl: Sclerosis. ~ Wilcoxon rank sum test, # Student’s t-test

Unadjusted *p*-value and adjusted, corrected q-value (for age, body mass, time from injury/deployment, exposure to trauma exposure, socio-economic status and ethnicity, and multiple testing using false discovery rate) Significant results highlighted in bold

## Discussion

This large unique cohort offers possible insights into the pathophysiological processes leading to the radiographic presentation of PTOA after severe trauma. The study shows that different markers, reflective of different molecular processes, potentially influence the post-traumatic onset of the three key radiographic features of OA, namely JSN, osteophytes and sclerosis. Cross-sectionally, leptin was significantly higher in those with JSN. Longitudinally, COMP offered some predictive value for the new development of JSN, with panels of COMP, IL-1β and leptin, and TNF-α, IL-1β and adiponectin doing the same for new osteophyte and sclerosis, respectively. These differing biomarkers could open a window into the underlying pathomechanisms contributing to the early PTOA structural changes that occur after trauma.

PTOA is of particular interest to researchers, due to presentation in a younger population, with fewer co-morbidities, and a clear initiating event. PTOA pathological processes likely represent a failure of initial injury repair and/or remodelling, and altered joint homeostasis with a resultant imbalance between anabolism and catabolism. It has an accelerated pathophysiological process, present within a few years [4], potentially accelerated further following major trauma (such as in military personnel) [3]. As a result, military individuals who have sustained significant injuries during combat are extremely high-risk, and are useful to study from a PTOA mechanistic point of view. Investigating individual radiological features can provide possible explanations of each pathway and therefore, an individual’s underlying endotype. A better understanding of endotypes, just like phenotypes, allows a personalised approach for interventions or improvements in drug discovery trial recruitment [14].

Recommended as a primary endpoint for structural change by the European Medicines Agency and the Federal Drugs Agency [31, 32], JSN is considered a proxy measure for depth, health, and integrity of hyaline articular cartilage. During the PTOA development, articular cartilage undergoes loss in tensile strength and pressure absorption, likely as a result of a mismatch between ECM catabolism and anabolism, and subsequent disruption of homeostasis, in the context of chronic inflammation [1, 9]. This study supports those findings with COMP seen to have an AUROC of 0.604 for new cases of JSN, and whilst the R2 was very low, as were the correlations seen in Figs. 1 and 2, the increase in COMP suggests increased, and unbalanced, catabolic action [33]. In addition, leptin is higher in those with JSN, an adipokine known to influence OA via an inflammatory mechanism influencing cartilage catabolism further [34].

Osteophytes, initially cartilaginous outgrowths which subsequently undergo bony ossification, can be an early sign of OA development, and are created in response to load to increase joint stability and compensate for physiological demand [35]. Joint malalignment, either due to biomechanical influence, co-existing disease, or joint injury, can be initially mitigated by osteophytes, commonly at joint margins, before pathological adaption occurs often due to increased instability or increased load (including body mass) [36]. The increased COMP and PIIANP levels for those with osteophytes at baseline support the role of ECM turnover in this process, though subsequent adjustment and correction made these differences non-significant. Three biomarkers, COMP, IL-1β and leptin, offered an AUROC of 0.586, with this finding, and the correlations in Figs. 1 and 2, further demonstrating the imbalance of cartilage and collagen catabolism and anabolism in the presence of inflammation.

Subchondral bone sclerosis can be overlooked in the PTOA process, especially given the focus on understanding the processes underpinning and driving articular cartilage degradation [37, 38]. The proinflammatory cascade, triggered by chondrocytes in response to cartilage damage, can lead to subchondral bone turnover and increased vascularisation, ending with bone marrow lesions and sclerosis [38]. However, subchondral bone itself can drive cartilage degeneration, with bone-generated cytokines (including IL-1β and-6) and growth factors (such as insulin-like growth factor-1), passing through the tidemark and driving cartilage metabolism [38], hence trabecular bone modelling is a validated marker of PTOA progression [39]. In addition, adipokines have been seen to be associated with remodelling in OA, and the formation of fibrosis in other conditions [40, 41], with post-traumatic fibrosis likely to explain this finding. This study found that the unadjusted adipokines, leptin and adiponectin were significantly higher and lower, respectively in those with sclerosis (non-significant post-adjustment/correction), with TNF-α, IL-1β and adiponectin offering an AUROC of 0.59 for new cases, providing an molecular insight into trabecular and subchondral bone remodelling.

It is important to contextualise these results. Given the R2 and correlation values, there is likely limited clinical applicability. However, what is notable is that these findings are all in a cohort in their 30’s, male, and with a predominance for early radiographic change, thus, these data offer novel insights into the complex PTOA pathophysiology. It is expected that, as the cohort progresses, the rates and severity will increase further (as has begun between baseline and follow-up, Table 3), offering further insight into PTOA processes. These initial results demonstrate that there are distinct pathophysiological processes at play for each component of PTOA structural change, hence different interventions, especially pharmacological ones, might be required for distinct structural endotypes, and subsequent phenotypes. Given the significant risk of PTOA, especially in those exposed to major trauma, then proactive interventions should be considered, and now different pathological processes have been identified using biomarker clusters, then these pathways should be considered for targeted multi-modal intervention. These results offer a foundation of understanding, which need to be better demarcated. This will be possible as more follow-up visits are completed in this large, unique cohort, with advanced analysis techniques, such as proteomic analysis to better understand underlying pathological processes [42]. This latter analysis is planned, using these findings as hypothesis-generating for specific pathway analysis, alongside an unbiased clustering approach.

There are strengths and limitations to this study. A key strength is the size of population (*n* = 872), and whilst the all-male study aged in their 30’s is a limitation, this is an under-represented population in OA research. Another strength of this work is the longitudinal nature of the data. Weaknesses include a lack of a replication cohort, the single radiographic view of the knee, which might contribute to underscoring of OA features, and the floor and ceiling effect of the serum biomarkers, which is likely to reduce the statistical power to detect stronger correlations with radiographic features. Furthermore, the biomarkers were only measured at single point in time, which limits their ability to predict change – further work is planned to mitigate this. Finally, the KOALA scoring system reports low specificity, particularly of JSN and sclerosis, which might influence results.

## Conclusion

This study reports the relationship between a panel of candidate ECM turnover, inflammatory and metabolic serum biomarkers and the individual radiographic features of knee OA in a large, young, male cohort over multiple timepoints. In line with the prespecified hypothesis, different clusters of biomarkers had relationships with each feature, suggesting different pathological processes at play, including predominant unbalanced ECM-catabolism in the presence of inflammation contributing to JSN, ECM-catabolism and increased inflammation contributing to osteophyte development and an inflammation-predominant process contributing to subchondral sclerosis.

## Supplementary Information

Below is the link to the electronic supplementary material.

Supplementary Material 1

## Data Availability

Data relate to serving and ex-serving military personnel, are sensitive and are not widely available, however, requests for data can be made via the corresponding author and will be considered on a case-by-case basis and subject to UK Ministry of Defence clearance. The code used for analysis will be shared on request to the corresponding author.
