# Supplementary material for: Insights into knee post-traumatic osteoarthritis pathophysiology from the relationship of serum biomarkers to radiographic features in the ADVANCE cohort
Source: Arthritis Res Ther. 2025 Nov 5;27:207. doi: 10.1186/s13075-025-03648-y (PMC12587722; doi:10.1186/s13075-025-03648-y)
Supplement: Supplementary file 1 — Supplementary Material 1 [file 13075_2025_3648_MOESM1_ESM.docx]

Supplementary file 1: Correlation analysis

|  | Cross-sectional (coefficient, p & q value) | | | Longitudinal (coefficient, p & q value) | | | | | |
| --- | --- | --- | --- | --- | --- | --- | --- | --- | --- |
|  | JSN | OP | Scl | New JSN | New OP | New Scl | Prog JSN | Prog OP | Prog Scl |
| IL-1β# | 0.0504  0.1386  0.2396 | 0.0266  0.4326  0.4944 | 0.0105  0.7577  0.8659 | 0.0359  0.4041  0.8082 | 0.0831’  0.0272  0.1144 | 0.1028’~  0.0036  0.0288 | 0.0588  0.286  0.7187 | -0.0396  0.6133  0.8755 | 0.0063  0.9576  0.9576 |
| TNF-α* | 0.0426  0.206  0.2396 | 0.0036  0.9157  0.9157 | 0.019  0.5749  0.7665 | -0.0141  0.7441  0.8504 | -0.0209  0.5784  0.6610 | -0.0714’  0.0436  0.1744 | 0.0619  0.2653  0.7187 | -0.0245  0.756  0.8755 | 0.0618  0.602  0.8762 |
| IL17-α* | 0.0425  0.2097  0.2396 | 0.0288  0.3955  0.4944 | 0.0548  0.1057  0.2114 | -0.0162  0.7077  0.8504 | 0.0385  0.3073  0.5216 | -0.0112  0.7524  0.7524 | 0.1364’  0.0116  0.0928 | -0.0123  0.8755  0.8755 | 0.1536  0.1919  0.7676 |
| CTX-II# | -0.0017  0.9592  0.9592 | 0.0333  0.3264  0.4944 | -0.0016  0.9613  0.9613 | 0.0028  0.9491  0.9491 | 0.0344  0.3609  0.5216 | -0.0125  0.724  0.7524 | -0.0375  0.4965  0.7187 | -0.0209  0.7896  0.8755 | -0.0601  0.6112  0.8762 |
| Leptin# | 0.1311’~  0.0001  0.0004 | 0.0934’~  0.0004  0.0016 | 0.1202’~  0.0004  0.0032 | 0.0705  0.1014  0.4052 | 0.0678  0.0717  0.1912 | 0.0421  0.235  0.47 | 0.0249  0.6521  0.7187 | 0.0975  0.2126  0.8755 | 0.0374  0.7518  0.8762 |
| COMP* | 0.0988’~  0.0035  0.014 | 0.1142’~  0.0007  0.0019 | 0.0421  0.2147  0.3435 | 0.1418’~  0.0009  0.0072 | 0.0823’  0.0286  0.1144 | 0.0251  0.48  0.64 | 0.0315  0.5727  0.7187 | 0.0532  0.4994  0.8755 | 0.0721  0.5427  0.8762 |
| Adipo* | -0.0724’  0.0326  0.0869 | -0.1235’~  0.0003  0.0016 | -0.1035’~  0.0022  0.0088 | -0.0519  0.2282  0.6085 | 0.004  0.9155  0.9155 | 0.0533  0.1326  0.3536 | -0.0201  0.7187  0.7187 | -0.0829  0.2913  0.8755 | -0.1811  0.1228  0.7676 |
| PIIANP* | -0.0442  0.1917  0.2397 | -0.0881’~  0.0092  0.0184 | -0.0709’  0.0363  0.0968 | 0.0202  0.6392  0.8504 | -0.0323  0.3912  0.5216 | -0.0324  0.3605  0.5768 | 0.0396  0.4775  0.7187 | -0.0494  0.5304  0.8755 | -0.0352  0.7667  0.8762 |

*IL: Interleukin, TNF: Tumour Necrosis Factor, CTX-II: C-terminal cross-linked telopeptide of type II collagen, COMP: cartilage oligomeric protein, PIIANP: N-propeptide of collagen IIA, Adipo: Adiponectin. JSN: Joint space narrowing, OP: osteophytes, Scl: Sclerosis* # Pearson’s pairwise correlation * Spearman’s correlation

Supplementary file 2: Regression models

New JSN

AUROC: 0.6035

SE: 0.0309

95% CI: 0.54297,0.66398

AIC: 526.72

BIC: 535.31

New osteophytes

AUROC: 0.5862

SE: 0.0310

95% CI: 0.52546, 0.64686

AIC: 588.03

BIC: 606.27

New sclerosis

AUROC:0.5896

SE: 0.0356

95% CI: 0.51990,0.65932

AIC: 467.14

BIC: 485.87
